# Coping With COVID: Does Prior Military Service Play a Role for Vietnam Veterans?

**DOI:** 10.1093/geroni/igab046.503

**Published:** 2021-12-17

**Authors:** Jeanne Stellman, Steven Stellman, Anica Kaiser, Avron Spiro, Brian Smith

**Affiliations:** 1 Mailman School of Public Health, Columbia University, Mailman School of Public Health, Columbia University New York, New York, United States; 2 Department of Epidemiology, Mailman School of Public Health Columbia University New York, New York, United States; 3 National Center for PTSD at VA Boston Healthcare System, Boston University School of Medicine, VA Boston Healthcare System Boston, Massachusetts, United States; 4 Boston University, Boston, Massachusetts, United States; 5 Women's Health Sciences Division, National Center for PTSD, VA Boston Healthcare System Boston, Massachusetts, United States

## Abstract

We investigated the impact of earlier military combat on ability to cope with the COVID-19 pandemic in 379 male Vietnam veterans who responded to surveys in 1984, 1998, and 2020. Combat exposure was scored with a validated scale, contrasting lowest tertile (8-15) vs. medium/high (16-40). About one-fourth of veterans (26%) reported that their military experience made it easier to cope with the pandemic, while over half (59%) said it had no effect. Medium/high-combat veterans were more likely to report that their military experience made coping easier (OR = 1.8, p = 0.03), but were less likely to report no effect of service on their coping than low-combat veterans (OR = 0.40, p<0.001). All 19 respondents (5%) who said military experience made coping more difficult were medium/high combat veterans. Military experience, and combat particularly, affected many of these veterans’ ability to cope with the pandemic decades after their service.

